# M. Daudet's Darwinism

**Published:** 1889-12-21

**Authors:** 


### M. DAUDET'S DARWINISM.

M. Alphonse Daudet, as we recently informed our readers, has been grievously bitten by the "Spirit of the Age," as that term is understood in France. Not content with being and remaining one of the most delightful story-tellers of his time: the creator of *Le Petit Chose*, *Jack*, *Tartarin*, *Delobelle*, *Numa Roumestan*, *Bompard*, and others, and the author of some of the most charming *contes* in the French language, he has incontinently conceived the idea of becoming a moralist, and a satiric moralist to boot. It is as if Mr. Andrew Lang, the most delightful Trifler in these islands, were suddenly seized with the desire to sit in the seat of Mr. Herbert Spencer, and is as utterly inconsistent with M. Daudet's actual character, as it was at variance with the character of his *Tartarin de Tarascon* to endeavour to become an Alpine explorer. Be this as it may, the fact remains that M. Daudet has, after some reflection, discovered that the world is decidedly going to the bad, and that the doctrines of the late Mr. Darwin, and "the brutal Saxon *formulæ*: 'The strong eat the weak,' and 'The survival of the fittest,'" are the *teterrima causa* of this deterioration. And, in order to bring this discovery home to the minds of a careless generation, he has indited a drama which he has called *La Lutte pour la Vie*, and which we are, of course, shortly to see on the English stage. We have already given our readers some idea of the Preface M. Daudet has recently attached to his production; and we think they will not take it amiss if we now endeavour to give them some idea of the play itself, more especially as a correspondent, judging us rather too hastily, has thought fit to plead the merits of M. Daudet as a novelist, or, as he more elegantly terms it, "a practitioner of the noble art of fiction," in mitigation of remarks that are directed solely to M. Daudet's defects as a pseudo-scientific philosopher and playwright.

But to our muttons. The representative of M. Daudet's Darwinism, and the protagonist of his drama, is M. Paul Astier, a deputy of thirty-two years of age. His dominant characteristics are an addiction to Darwinism (of whose tenets, as M. Daudet ingenuously informs us, he knows nothing from actual reading); and, in consequence of his Darwinism, a cold, calculating, heartless "ambition," which is only restrained from actual crime by his "taste for power"—"a preservative," as M. Daudet says, probably with the example of M. Boulanger before his eyes, "a preservative as sure as *the best* conscience of an honest man!" Before he is introduced to us, he had induced a foolish, fond, and wealthy old Corsican duchess, whose age is given, with the pitiless precision of Science, at fifty, to pay his debts and to marry him. As he is a "*Darwiniste*," he, of course, endeavours to increase the gains he had acquired by his marriage by speculation. But his speculations fail; and just as he is on the verge of ruin, and is heartily sick of his devoted and loving, but ancient wife, whose fortune he has thus squandered, he comes across a young and beautiful Austrian Jewess, aged 20, "formidably rich," and a "Darwiniste," like himself. Of course, when two persons of different genders meet under such circumstances the Law of Sexual Selection comes into operation; and Paul Astier and Esther de Sélény form no exception to the general rule. Their mutual bad qualities—for Esther is quite as hard and heartless as Paul—instantly attract them to each other. They find in these qualities what Goethe would call their "elective affinity," and their determination to strengthen it by marriage is simultaneous. Paul Astier, therefore, endeavours to induce his wife to consent to a divorce; but as his Private Secretary has secretly informed her of her husband's motives for this divorce, she resolutely refuses to lend herself to the arrangement. Meanwhile, Paul Astier and Esther de Sélény continue to meet and to "flirt" (as they call it) at the opera of an evening and the Bois de Boulogne of a morning; and, in order to divert his wife's attention from his intrigue with Esther and to afford her a more undeniable pretext for divorce, Paul deliberately seduces a young girl, aged twenty, who is on the eve of being married to a doctor, aged twenty-five, who "adores" her and whom she equally "adores"; and when chance throws into his hands a bottle of strychnine with which his victim has poisoned herself, he maunders over the idea, which it suggests to him, of murdering his wife in a manner that may, perhaps, remind one of Macbeth and the dagger that he saw before him, but of a modern French Macbeth and absinthe. While he is thus hesitating at crime, his wife comes in, overhears his soliloquy, and in order, apparently, to move him to remorse, prepares a scene which recalls the famous play in Hamlet of the murder of the King of Denmark—but at a long interval, and with a decided difference. She gives a brilliant evening party to which she invites all her friends, and at which an "illustrious novelist" reads a realistic story dealing with a "Darwinian" murder not unlike that which Paul Astier meditates; and, while all her guests are engrossed in the details of this story, she deliberately puts herself in her husband's way and affords him the chance he has been vaguely wishing for—by asking him for a glass of water! This unexpected request simply "terrifies" Paul Astier. For a moment he stands speechless. Then, promptly returning to his Darwinian self, he rushes off the stage for the glass of water, his wife meanwhile watching him through the half-open door and sadly murmuring to herself: "Oh, the poor mother of Cain!" When Paul returns with the poisoned water, his wife takes the tumbler from his hand, plays with it awhile, puts it up to her mouth, puts it down again, and so on *da capo*; until at last, Paul, in an agony of some indefinable sentiment utterly unworthy of a "Darwiniste," probably a prevision of his "preservative," adjures her not to drink the water. "Then," says M. Daudet, taking up his parable in his Preface, "her heart overflows with the maternal pity that watches behind the love of every woman." She scolds him very much as a good mother would a bad son; then forgives him; and finally consents to the desired divorce. Six months afterwards her country estates are brought to the hammer, and Paul goes down to the auction, where he meets his beautiful "Darwiniste," who instantly accepts him. Just as the auctioneer is about to knock down to him a pair of bays, belonging to his old wife, which he has bought for the new one, M. Vaillant, aged sixty, the father of his victim, steps up to him, and says: "We struggle for existence, don't we? young man. The strong eats the weak. Then," and he produces a pistol, "I suppress you, bandit." So saying he fires. Paul falls down dead at Esther's feet. The auctioneer finishes his sentence: "Gone!" And M. Vaillant, with a grand gesture towards heaven, repeats it: "Gone! That's exactly the word!" And the curtain falls.

"Brave old Vaillant!" exclaims M. Daudet, who acts as a kind of Greek chorus to his own play. "He, at least, is not a '*struggle-for-lifeur*'; he comes of an old, a very old boat, in which they believed in a lot of things long since gone out of fashion." And he then confides to us that he has "in his blood such a hatred for the filthy beast"—Paul Astier, to wit, and his like—"that he would have been capable of shooting him himself." There is something irresistibly comic in the idea of an author conceiving a character for whom, as he grows under his hand, he contracts a greater and greater aversion; and, when he has finally put a violent end to his hateful creation, saying: "Ah! you filthy beast! take that!" Tacitus, when drawing Tiberius, and Mr. Kinglake, when laying his black strokes on the character of Napoleon III., are nothing in comparison with M. Alphonse Daudet. Whatever he may say or think about the "infamous theories of Darwin," he is determined that they shall not prevail, at any rate, not as long as he can draw a trigger or write a play. And this is a consolation—as far as it goes.

Such, stripped of its accessories, is *La Lutte pour la Vie*; and the accessories are like unto the principal agents thereof. There is, for instance, a serio-comic Italian Count, aged twenty-eight, who, probably owing to the present unsatisfactory relations between France and Italy, murders French in a manner that no educated Italian gentleman would do nowadays, and who is made to exclaim automatically whenever he sees a woman, "*Cristo! qu'elle est bella!*" Then there is the widow, aged forty, of an Austrian field-marshal (another unsatisfactory friend of France!), who "eternally" laments her late husband (who was a brute), and makes the most outrageous love to every man she comes across. Then there is a doctor, aged twenty-five, who was engaged to Paul Astier's victim, and who, in order, perhaps, to accentuate his inferiority to the "Darwiniste" is made to stutter at stated intervals, "*le—le—enfin.*" Then there is an unscrupulous lawyer's clerk, two years younger than Paul Astier, and an intimate friend of his, who seeks to push his fortunes, like his friend, by a rich marriage. And, lastly, there is the Private Secretary, aged twenty-three, who belongs, says M. Daudet, "to a boat which is coming after the 'Darwinian,' unballasted of all respect and of morality." This young gentleman is a follower of *Berckley*—by whom is intended our most excellent Bishop of Cloyne of two hundred years ago!—and he expresses himself as follows: "The *Scotch* doctrine, you know. Nothing exists, the world is a phantasmagoria! This principle once admitted, you can permit yourself anything; it is not of the very slightest importance. That's my theory." So then Berkeley was a Sotchman, not an Irishman; and, infamous as Darwin's doctrines are, the Bishop's are still worse, and will eventually prevail! No wonder that Lord Tennyson exclaims, in despair:

> "Chaos, Cosmos! Cosmos, Chaos! *who* can tell how all will end!"

"And this," said Macaulay, on a memorable occasion, "and this is fine poetry? This is what ranks its author with the master spirits of the age!" And this, we may repeat, is what is styled drama in "this century which calls itself the Nineteenth!" This is what the country of Molière now sends over to the land of Shakespeare! This is what will shortly be exercising the soft brains of our "Mashers" and "Best Young Men;" and offering fresh suggestions to the frivolous fashionable women who are their *raison d'etre*! Our correspondent, who displays a remarkable acquaintance with M. Daudet's motives, says that this play is due to "Daudet's opinion," that M. Zola's and kindred novels "have had an injurious effect on his fellow-countrymen." As, however, it is generally supposed that M. Zola has had "an injurious effect" on M. Daudet himself, perhaps "F" will be so good as to substantiate his statement. "*Si quid novit rectius istis*——"

